# Differential Inhibitory Effects of CysLT_1_ Receptor Antagonists on P2Y_6_ Receptor-Mediated Signaling and Ion Transport in Human Bronchial Epithelia

**DOI:** 10.1371/journal.pone.0022363

**Published:** 2011-07-22

**Authors:** Wendy Ka-hoi Lau, Alison Wai-ming Chow, Simon Chak-leung Au, Wing-hung Ko

**Affiliations:** School of Biomedical Sciences, The Chinese University of Hong Kong, Hong Kong, China; University of Las Palmas de Gran Canaria, Spain

## Abstract

**Background:**

Cysteinyl leukotriene (CysLT) is one of the proinflammatory mediators released by the bronchi during inflammation. CysLTs exert their biological effects via specific G-protein-coupled receptors. CysLT_1_ receptor antagonists are available for clinical use for the treatment of asthma. Recently, crosstalk between CysLT_1_ and P2Y_6_ receptors has been delineated. P2Y receptors are expressed in apical and/or basolateral membranes of virtually all polarized epithelia to control the transport of fluid and electrolytes. Previous research suggests that CysLT_1_ receptor antagonists inhibit the effects of nucleotides acting at P2Y receptors. However, the detailed molecular mechanism underlying the inhibition remains unresolved.

**Methodology/Principal Findings:**

In this study, western blot analysis confirmed that both CysLT_1_ and P2Y_6_ receptors were expressed in the human bronchial epithelial cell line 16HBE14o-. All three CysLT_1_ antagonists inhibited the uridine diphosphate (UDP)-evoked *I_SC_*, but only montelukast inhibited the UDP-evoked [Ca^2+^]_i_ increase. In the presence of forskolin or 8-bromoadenosine 3′5′ cyclic monophosphate (8-Br-cAMP), the UDP-induced *I_SC_* was potentiated but was reduced by pranlukast and zafirlukast but not montelukast. Pranlukast inhibited the UDP-evoked *I_SC_* potentiated by an Epac activator, 8-(4-Chlorophenylthio)-2′-O-methyladenosine-3′,5′-cyclic monophosphate (8-CPT-2′-*O*-Me-cAMP), while montelukast and zafirlukast had no such effect. Pranlukast inhibited the real-time increase in cAMP changes activated by 8-CPT-2′-*O*-Me-cAMP as monitored by fluorescence resonance energy transfer imaging. Zafirlukast inhibited the UDP-induced *I_SC_* potentiated by N^6^- Phenyladenosine- 3′, 5′- cyclic monophosphorothioate, Sp- isomer (Sp-6-Phe-cAMP; a PKA activator) and UDP-activated PKA activity.

**Conclusions/Significance:**

In summary, our data strongly suggest for the first time that in human airway epithelia, the three specific CysLT_1_ receptor antagonists exert differential inhibitory effects on P2Y_6_ receptor-coupled Ca^2+^ signaling pathways and the potentiating effect on *I_SC_* mediated by cAMP and Epac, leading to the modulation of ion transport activities across the epithelia.

## Introduction

Bronchial asthma is an inflammatory disease that affects millions of people worldwide. Among the proinflammatory mediators released by the bronchi are cysteinyl leukotrienes (CysLTs). They are lipid mediators derived from arachidonic acid by the 5-lipoxygenase (5-LO) pathway [1] and play critical roles in the pathogenesis of asthma [2], [3]. CysLTs exert their biological effects via specific G-protein-coupled receptors. To date, there are two cloned human CysLT receptor subtypes, namely CysLT_1_ and CysLT_2_ [4]. Specific CysLT_1_ receptor antagonists, such as montelukast, pranlukast, and zafirlukast, are available for clinical use for the treatment of asthma [5]. Recently, crosstalk between CysLT_1_ receptor and P2Y_6_ receptor signaling systems has been reported in human mast cells [6] and monocyte/macrophage-like cells [7]. P2Y_6_ is a member of the P2Y receptor family that is expressed in the apical and/or basolateral membranes of virtually all polarized epithelia to control the transport of fluid and electrolytes [8], [9]. It has been shown that uridine diphosphate (UDP), a selective agonist for the P2Y_6_ receptor, can also activate the CysLT_1_ receptor. On the other hand, CysLT_1_ receptor antagonists could inhibit the effects of the extracellular nucleotide acting at P2Y receptors [10]. Evidence also suggests that CysLTs and UDP do not share the same receptor and the CysLT_1_ receptor possesses dual CysLT/UDP specificity.

In the airway, chloride (Cl^−^) secretion and sodium (Na^+^) reabsorption can be modulated by the activation of multiple P2Y receptors that couple to the phospholipase C (PLC) and calcium-signaling pathway. Recently, work from this laboratory has confirmed that a human bronchial epithelial cell line, 16HBE14o-, expresses multiple P2Y receptors mRNA and proteins, including the P2Y_6_ receptor [11]. UDP could stimulate both calcium (Ca^2+^)- and 3′,5′-cyclic monophosphate (cAMP)-dependent chloride ion secretion in 16HBE14o- cells. An increase in cAMP production could in turn activate both protein kinase A (PKA) and an exchange protein directly activated by cAMP (Epac) [12]. As there is little knowledge regarding the effects of specific CysLT_1_ receptor antagonists on airway epithelial transport, the aim of this project was to examine their effects on P2Y_6_ receptor-mediated Cl^−^ secretion in a human bronchial epithelial cell line (16HBE14o-) and to investigate the possible signal transduction pathway(s) through which the antagonists may act.

## Results

### Expression of CysLT_1_ and P2Y_6_ receptors in the 16HBE14o- cell monolayer

**Figure 1:**
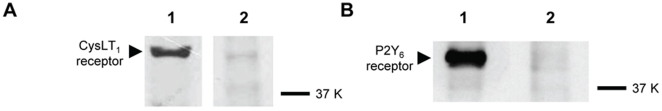
Western blotting analysis showing the protein expression of CysLT_1_ and P2Y_6_ receptors in 16HBE14o- cells. The expression of CysLT_1_ (44 kDa) and P2Y_6_ (41 kDa) receptors was demonstrated (lane 1), and their positions on the blot closely matched their calculated molecular masses of 39 kDa and 36 kDa, respectively. Detection of these protein bands appeared specific, as they were mostly blocked by prior reabsorption of the antibodies with their respective control antigen for 2 h at 4°C (lane 2).

To examine the presence of CysLT_1_ and P2Y_6_ receptors in 16HBE14o- cells, western blot analysis was conducted. The protein expression of CysLT_1_ and P2Y_6_ receptors in 16HBE14o- cells was detected as shown in Fig. 1. The CysLT_1_ receptor polyclonal antibody identified an intense 44-kDa band in whole cell lysates of 16HBE14o- cell monolayers (Fig. 1A, left lane). The specificity of the band was confirmed by the complete abolishment of the immunoreactive signal in 16HBE4o- cells by the CysLT_1_ receptor polyclonal antibody that had been preadsorbed with specific blocking peptides. The blocking peptides correspond to amino acid residues 318–337 of the human CysLT_1_ receptor (Fig. 1A, right lane). On the other hand, the P2Y_6_ receptor was identified as an intense 41-kDa band (Fig. 1B, left lane). The specificity of the band was confirmed by the complete abolishment of the immunoreactive signal by the P2Y_6_ receptor antibody preadsorbed with specific blocking peptides. The blocking peptides correspond to amino acid residues 322–343 of the human P2Y_6_ receptor (Fig. 1B, right lane). Western blot analysis demonstrated that 16HBE14o- cells expressed CysLT_1_ receptors and confirmed our previous finding on P2Y_6_ receptor expression in this cell line [11].

### Activation of the P2Y_6_ but not the CysLT_1_ receptor stimulated increases in *I_SC_* and [Ca^2+^]_i_ in 16HBE14o- cells

**Figure 2:**
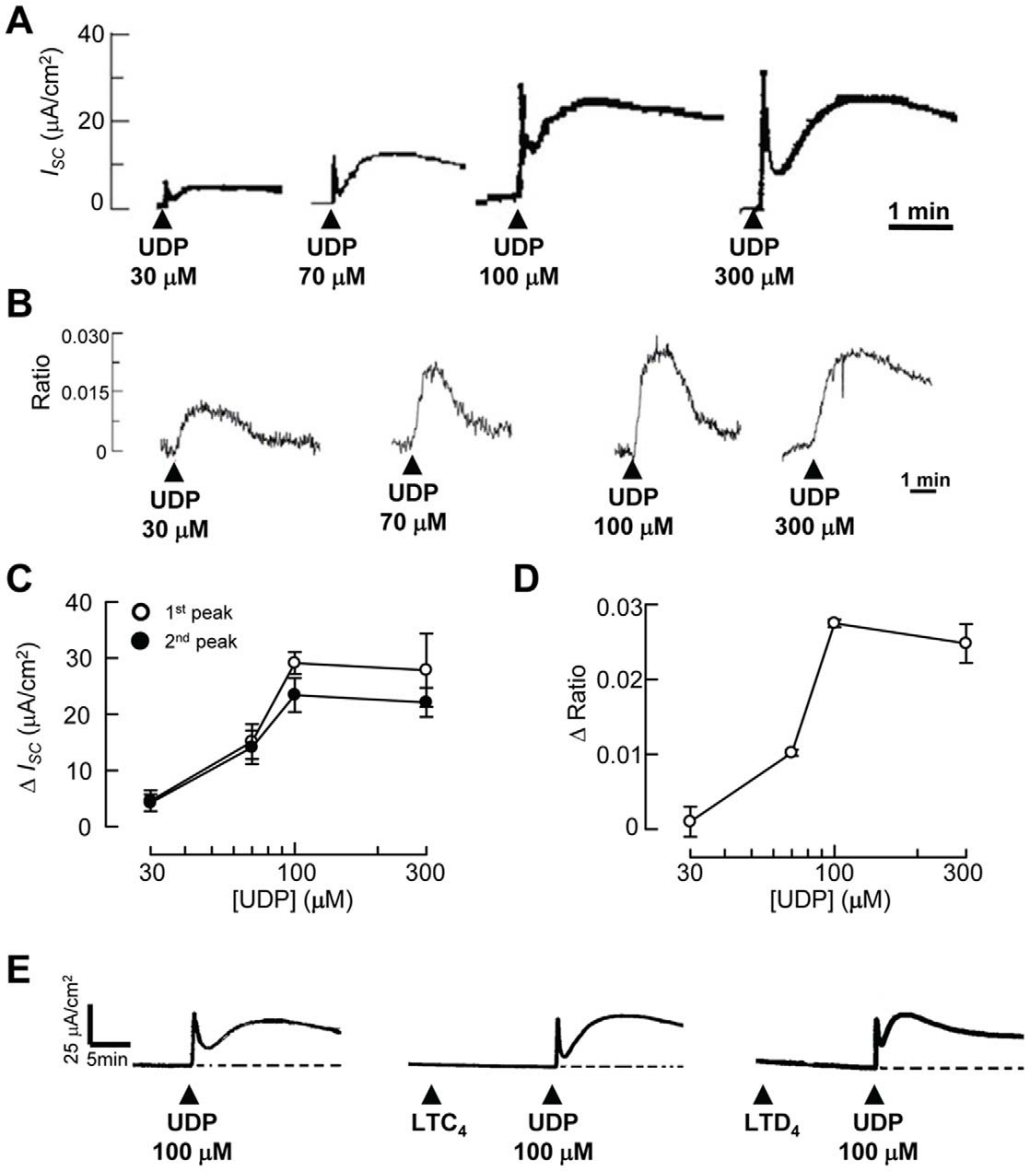
Concentration-dependent effect of UDP on *I_SC_* and [Ca^2+^]_i_. (A) Epithelia were initially bathed in normal K-H solution while changes in *I_SC_* were recorded. A serosal-to-mucosal-directed Cl^−^ gradient was applied across the monolayers by changing the apical K-H solution to one with reduced Cl^−^ concentration to facilitate Cl^−^ secretion (not shown). Different concentrations of UDP were added to the apical side as indicated. Data presented are representative of at least four independent experiments. (B) UDP-evoked increases in [Ca^2+^]_i_ were monitored by a microfluorimetric technique using the calcium-sensitive fluorescent dye Fura-2. Cells grown on glass coverslips were initially superfused with normal K-H solution. The K-H solution was then changed to a nominally Ca^2+^-free K-H solution, and the cells were stimulated with different concentrations of UDP as indicated. Data presented are representative of at least four independent experiments. (C) Concentration-response relationship for the effect of UDP upon Δ*I_SC_* in 16HBE14o- epithelia. The changes in *I_SC_* for the first and second peaks were quantified and plotted against the concentration of UDP used. Each data point represents the mean ± S.E. (*n* = 4–5). (D) Concentration-response relationship for the effect of UDP upon changes in [Ca^2+^]_i_ represented by Δratio. Each data point represents the mean ± S.E. (*n* = 4–5). (E) Application of CysLT_1_ receptor agonists (10 µM), LTC_4_ or LTD_4_, did not cause any discernible increase in *I_SC_* and did not affect the subsequent 100-µM UDP-evoked *I_SC_* responses. Data presented are representative of at least four independent experiments.

UDP is the specific agonist of the P2Y_6_ receptor [13], [14], which has been shown to regulate ion transport processes in airway epithelial cells via both Ca^2+^- and cAMP-dependent pathways [11]. First, the effect of apical application of UDP on *I_SC_* was investigated using a conventional Ussing chamber study. Fig. 2A shows the representative recordings of *I_SC_* in response to the apical application of 30–300 µM UDP. The addition of UDP to the apical side rapidly increased *I_SC_*. The *I_SC_* change consisted of biphasic responses, namely an initial transient peak (first peak) and a second increase within 2 min (second peak). In another series of experiments, the intracellular calcium concentration was measured in 16HBE14o- cells grown on glass coverslips by using a fluorescent technique. Fig. 2B shows the representative recordings of [Ca^2+^]_i_ in response to 30–300 µM UDP application. The changes in [Ca^2+^]_i_ were obtained by measuring the maximal increase of the 340/380 nm fluorescence ratio upon stimulation.

Fig. 2C and 2D summarize the change of *I_SC_* (Δ*I_SC_*) and fluorescence ratio (Δratio) induced by UDP application, respectively. Apical application of UDP elicited a concentration-dependent increase in the first peak of *I_SC_*, with a UDP concentration of 100 µM eliciting the maximal increase in *I_SC_*. The increase in the second peak of *I_SC_* also exhibited a concentration-dependent relationship with the apical application of UDP, again with 100 µM UDP eliciting the maximal increase in *I_SC_*. The addition of basolateral UDP evoked only a small and variable increase in *I_SC_* in both phases (data not shown). Due to the small response of basolateral UDP-induced *I_SC_*, only *I_SC_* induced by apical addition of UDP was investigated further. The increase in [Ca^2+^]_i_ upon the addition of UDP also exhibited concentration dependency, again with 100 µM UDP inducing the largest increase in [Ca^2+^]_i_. In our previous study, the UDP-induced [Ca^2+^]_i_ increase was abolished and the increase in *I_SC_* response was greatly attenuated when the cells were treated with BAPTA-AM, an intracellular Ca^2+^ chelator. The results therefore confirmed our previous findings that UDP could stimulate a calcium-dependent *I_SC_* in 16HBE14o- cells [11].

Although CysLT_1_ receptor protein was expressed in 16HBE14o- cells, activation of the receptor by its agonists, CysLT C_4_ (LTC_4_) or LTD_4_ [15], did not stimulate any discernible increase in [Ca^2+^]_i_ and had no effect on the subsequent increase in Fura-2 ratio induced by UDP (UDP alone: 0.060±0.004; +LTC_4_: 0.049±0.005; +LTD_4_: 0.052±0.004, *p*>0.05). As shown in Fig. 2E, the presence of LTC_4_ or LTD_4_ also did not affect the basal *I_SC_* and the subsequent UDP-evoked *I_SC_* (UDP alone: 29.3±2.1 µA/cm^2^; +LTC4: 22.4±2.4 µA/cm^2^; +LTD4: 21.6±4.8 µA/cm^2^, *n* = 6, *p*>0.05). It appears that 16HBE14o- cells do not possess functional CysLT_1_ receptors that couple to the calcium-signaling pathway and thus did not exhibit any changes in *I_SC_*.

### CysLT_1_ receptor antagonists inhibited UDP-evoked *I_SC_*

**Figure 3:**
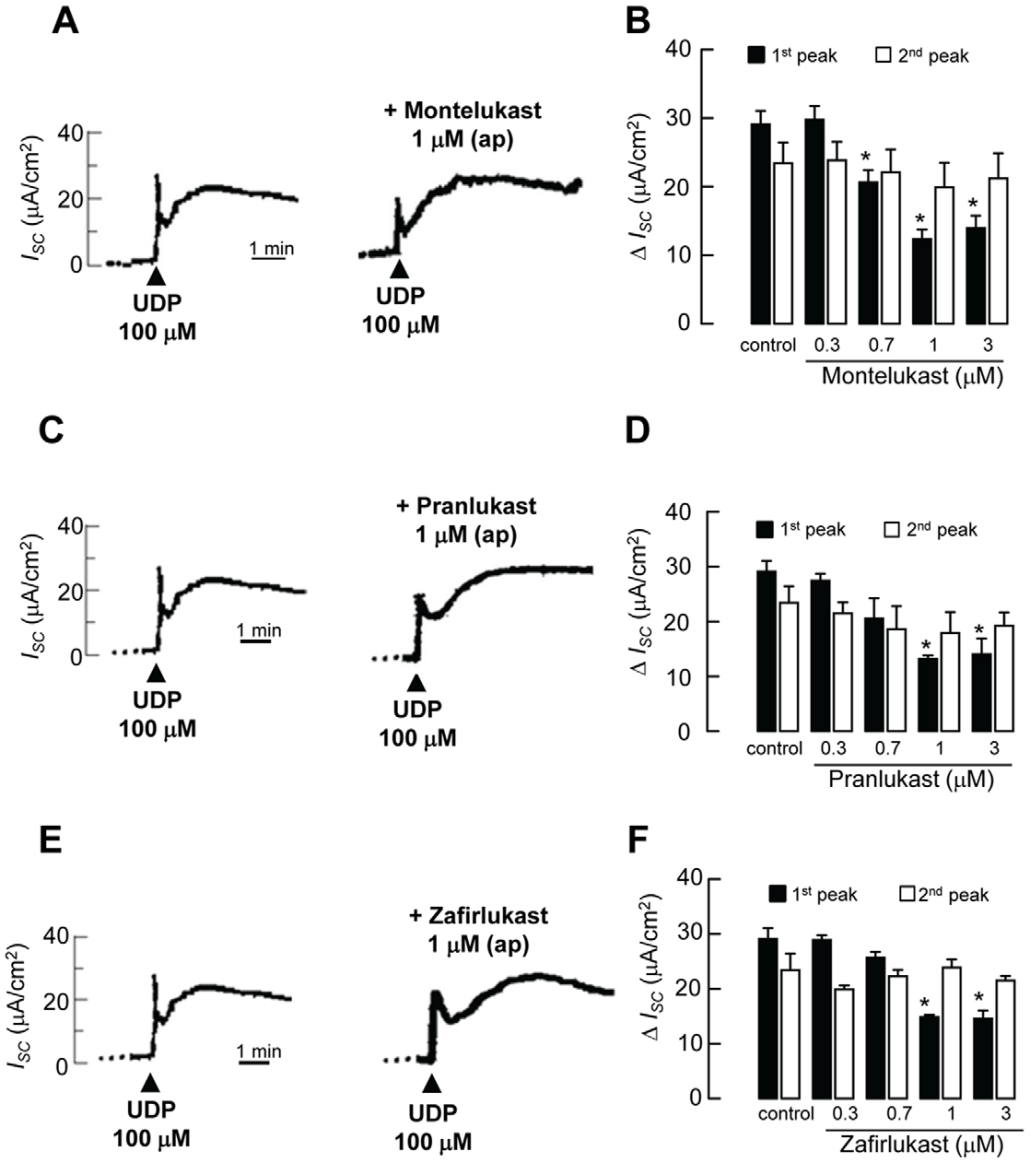
Effect of CysLT_1_ receptor antagonists, montelukast, pranlukast, and zafirlukast, on UDP-evoked *I_SC_* responses. Representative recordings of *I_SC_* in response to the apical application of 100 µM UDP (shown by arrows) in the presence of montelukast (A). The CysLT_1_ receptor antagonist was applied 10 min before the application of UDP (data not shown). Data are summarized for the inhibitory effects of montelukast (B) on the first and second peaks of *I_SC_* increases in response to UDP. Similar experiments were performed using pranlukast (C and D) or zafirlukast (E and F). The control was the apical application of UDP alone in the absence of any CysLT_1_ receptor antagonist. Each column represents the mean ± S.E. (*n* = 4–5). (*, *p*<0.05, one-way ANOVA with Bonferroni post-hoc test compared with control).

The functional relationships between the CysLT and P2Y receptors have been highlighted in several recent reports. For example, it has been shown that CysLT_1_ receptor antagonists inhibit the effects of nucleotides acting at P2Y receptors in U937 cells [10]. Therefore, the effect of three commonly used CysLT_1_ antagonists (montelukast, pranlukast, and zafirlukast) on P2Y_6_ receptor-coupled *I_SC_* was measured in 16HBE14o- cells. The epithelia were pretreated with various concentrations (0.3–3 µM) of CysLT_1_ antagonists at the apical side, followed by apical application of 100 µM UDP. Addition of montelukast, pranlukast or zafirlukast (up to 3 µM) alone did not stimulate any increase in *I_SC_*. Fig. 3A shows the representative recordings of *I_SC_* in response to the application of 100 µM UDP in the absence (control) or presence of montelukast (1 µM). Fig. 3B summarizes the changes of the first and second peaks of *I_SC_* in response to the application of 100 µM UDP after apical pretreatment with montelukast. In the presence of apical montelukast (0.7, 1, and 3 µM), the UDP-induced first peak was significantly reduced. The second peak of *I_SC_* was not affected by montelukast.

Similar responses were obtained when the epithelia were stimulated with UDP in the presence of pranlukast (Fig. 3C) or zafirlukast (Fig. 3E). The UDP-induced first *I_SC_* peak was significantly inhibited by apical pretreatment of the epithelia with 1 and 3 µM pranlukast (Fig. 3D) or zafirlukast (Fig. 3F). Again, the UDP-induced second *I_SC_* peak was not inhibited by the apical application of pranlukast or zafirlukast. For the highest concentration of CysLT_1_ receptor antagonists tested (3 µM), approximately 50% of the UDP-evoked first peak of *I_SC_* was inhibited. All three CysLT_1_ receptor antagonists failed to inhibit the apical UDP-evoked *I_SC_* (first and second peaks) when applied to the basolateral aspect of the epithelia (data not shown). Similar experiments were conducted using a P2Y_2_/P2Y_4_ agonist, UTP (100 µM). Interestingly, all three CysLT_1_ receptor antagonists (1 µM) failed to inhibit the UTP (P2Y_2_/P2Y_4_)-mediated increase in *I_SC_* (control: 9.7±0.9 µA/cm^2^; +montelukast: 8.6±1.8 µA/cm^2^; +pranlukast: 8.8±1.0 µA/cm^2^; +zafirlukast: 9.2±1.3 µA/cm^2^, *n* = 5, *p*>0.05). Although P2Y_1_ receptors are expressed in 16HBE14o- cells [11], their specific agonist 2-methio- adenosine diphosphate (100 µM) failed to elicit any increase in *I_SC_* (*n* = 4).

### Montelukast, but not pranlukast and zafirlukast, inhibited UDP-evoked [Ca^2+^]_i_ increase in 16HBE14o- cells

**Figure 4:**
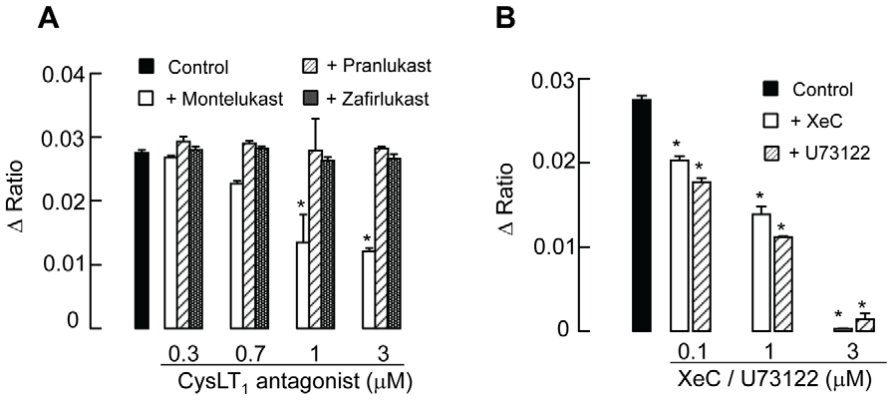
Effect of CysLT_1_ receptor antagonists, montelukast, pranlukast, and zafirlukast on UDP-evoked [Ca^2+^]_i_ responses. (A) Cells were stimulated with UDP in the absence (control) or presence of different concentrations of CysLT_1_ receptor antagonists applied 10 min prior to the addition of UDP (100 µM). (B) The UDP-evoked [Ca^2+^]_i_ responses were inhibited by different concentrations of an IP_3_ receptor antagonist, XeC, or a PLC inhibitor, U73122. Each column represents the mean ± S.E. (*n* = 4–6). (*, *p*<0.05, one-way ANOVA with Bonferroni post-hoc test compared with control).

Because UDP could elicit a calcium-dependent increase in *I_SC_*, we tested whether the three CysLT_1_ antagonists could inhibit UDP-evoked calcium signals. Addition of montelukast, pranlukast or zafirlukast (up to 3 µM) alone did not stimulate any increase in [Ca^2+^]_i_. The UDP-induced [Ca^2+^]_i_ was reduced by 17.5±2.2%, 50.9±2.9%, and 56.1±3.4% in the presence of 0.7, 1, and 3 µM montelukast, respectively (Fig. 4A). In contrast to the effect of montelukast, the UDP-induced [Ca^2+^]_i_ was not significantly reduced by pranlukast and zafirlukast up to 3 µM. Therefore, although all three CysLT_1_ antagonists could inhibit UDP-evoked *I_SC_*, pranlukast and zafirlukast failed to cause any inhibitory effects on calcium signals evoked by UDP. Similar experiments were conducted using the P2Y_2_/P2Y_4_ agonist UTP (100 µM) to stimulate the cells. All three CysLT_1_ receptor antagonists (1 µM) failed to inhibit the UTP (P2Y_2_/P2Y_4_)-mediated increase in [Ca^2+^]_i_ (control: 0.015±0.001 unit; +montelukast: 0.013±0.001 unit; +pranlukast: 0.016±0.001 unit; +zafirlukast: 0.017±0.001 unit, *n* = 5, *p*>0.05). The P2Y_1_ receptor agonist 2-methio-adenosine diphosphate (100 µM) did not cause any discernible increase in [Ca^2+^]_i_ (*n* = 4).

The increase in [Ca^2+^]_i_ evoked by UDP is caused by the coupling of P2Y_6_ receptors to PLC and hence the production of the inositol trisphosphate (IP_3_) [13]. To test the effect of montelukast on P2Y_6_ receptor-dependent calcium signaling, two pharmacological agents, U73122 and xestospongin C (XeC), were used. U73122 is a specific inhibitor of PLC [16], whereas XeC is a novel blocker of the IP_3_ receptor [17]. In the experiment, the cells were perfused with normal K-H solution containing 2.5 mM Ca^2+^, which was then changed to a nominally Ca^2+^-free K-H solution. XeC in various concentrations was added 10 min before the application of 100 µM UDP. Under the pretreatment of the epithelia with 0.1, 1 or 3 µM XeC, the UDP-induced [Ca^2+^]_i_ response was reduced to 73.7±1.7, 50.6±3.5, and 1.1±1.0% of the control response (Fig. 4B), respectively. Similarly, U73122 (0.1, 1, 3 µM) reduced the UDP-induced [Ca^2+^]_i_ response to 64.4±3.5, 40.7±5.6, and 5.2±2.1% of the control response, respectively. The data demonstrated that P2Y_6_ receptors are coupled to the PLC/IP_3_ signaling pathway, leading to an increase in [Ca^2+^]_i_.

To test whether montelukast could interfere with the PLC/IP_3_ signaling pathway, experiments were performed by coincubation of the 16HBE14o- cells with both montelukast (1 µM) and XeC (0.1 µM). In the presence of montelukast and XeC, the UDP-induced [Ca^2+^]_i_ response was reduced to 71.9±3.2% of the control response, which is not statistically different from the response in the presence of XeC alone (*n* = 6, *p*>0.05). Therefore, coincubation of XeC and montelukast failed to cause additional inhibition of the UDP-induced [Ca^2+^]_i_ increase. The data suggest that montelukast may interfere with the signaling pathway at a level upstream of the IP_3_-induced Ca^2+^ release. To investigate whether montelukast may act upstream of the IP_3_-induced Ca^2+^ release, the epithelia were coincubated with both U73122 (0.1 µM) and montelukast (1 µM). Similarly, the data demonstrated that coincubation of the epithelia with both U73122 and montelukast reduced the UDP-induced [Ca^2+^]_i_ response to 63.2±2.6% of the control response, which is not statistically different from that in the presence of U73122 alone (*n* = 5, *p*>0.05). Taken together, the data suggest that montelukast may interfere with the calcium-signaling pathway at a level upstream of PLC activity and the production of IP_3_.

### CysLT_1_ antagonists inhibited the UDP-evoked Cl^−^ secretion in the presence of cAMP elevating agents, Epac or PKA activator

It has been shown recently by several studies that activation of P2Y_6_ receptors leads to the activation of Ca^2+^- and cAMP-dependent Cl^−^ secretion across airway epithelial cells [11], [18], [19], possibly by increasing cellular cAMP production [11], [20]. Increases in cAMP production could in turn activate two downstream signaling molecules, PKA and exchange protein directly activated by cAMP (Epac) [12], which could then regulate epithelial Cl^−^ secretion [21]. In this study, the effect of CysLT_1_ receptor antagonists on the potentiating effect of cAMP-elevating agents (forskolin, 8-Br-cAMP) and specific Epac [8-(4-Chlorophenylthio)-2′-O-methyladenosine-3′,5′-cyclic monophosphate; 8-CPT-2′*O*-Me–cAMP] and PKA [N^6^- Phenyladenosine- 3′, 5′- cyclic monophosphorothioate, Sp- isomer; Sp-6-Phe-cAMP] activators was examined. The potentiator was added about 10 min prior to the application of UDP.

**Figure 5:**
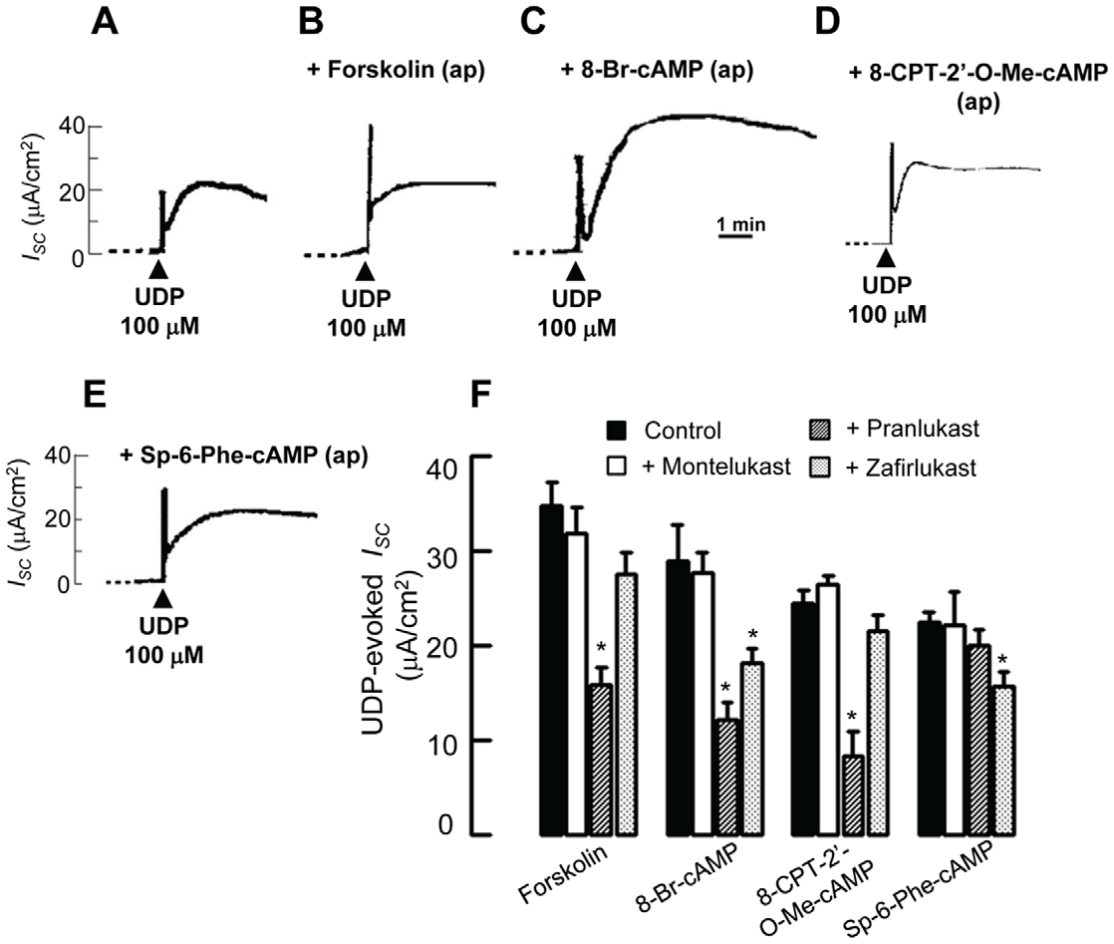
Inhibition by CysLT_1_ receptor antagonists of the potentiating effect of cAMP and Epac on UDP-evoked *I_SC_*. Representative recordings of *I_SC_* showing the response to the apical application of 100 µM UDP alone (A) or in combination with pretreatment of the epithelia with 1 µM forskolin (B), 8-Br-cAMP (C), 8-CPT-2′-*O*-Me-cAMP (D), or Sp-6-Phe-cAMP (E) 10 min prior to the application of UDP. (F) Summarized data showing the potentiating effect of cAMP or Epac on UDP-evoked *I_SC_* could be inhibited by different CysLT_1_ receptor antagonists. Control refers to the *I_SC_* responses of the epithelia to UDP in the presence of various potentiating agents. Each column represents the mean ± S.E. (*n* = 4–6). (*, *p*<0.05, one-way ANOVA with Bonferroni post-hoc test compared with control).

In the first series of experiments, general cAMP elevating agents, such as forskolin and 8-Br-cAMP were employed. As shown in Fig. 5, forskolin (1 µM) greatly potentiated both the first and second peaks of *I_SC_* induced by UDP. The percentage increases of the first and second peaks were 198.0±9.2% (UDP: 17.50±1.95 µA/cm^2^; +Forskolin: 34.75±2.40 µA/cm^2^, *p<0.05*, *n* = 4–6) and 120.0±13.4% (UDP: 21.30±3.90 µA/cm^2^; +Forskolin: 25.60±2.85 µA/cm^2^, *p<0.05*, *n* = 4–6), respectively. Similarly, 8-Br-cAMP, a cAMP analog, potentiated the first and second peaks of *I_SC_* to 165.54±13.78% (+8-Br-cAMP: 28.96±3.80 µA/cm^2^, *p<0.05*, *n* = 4–6) and 180.5±28.80% (+8-Br-cAMP: 38.45±6.13 µA/cm^2^, *p<0.05*, *n* = 4–6), respectively.

Forskolin and 8-Br-cAMP are general cAMP level elevators that cannot distinguish between their two downstream targets, PKA and Epac. Therefore, in the second series of experiments, specific Epac or PKA activator were used. 8-CPT-2′-*O*-Me-cAMP and Sp-6-Phe-cAMPs were thus employed to potentiate the UDP-induced *I_SC_*. 8-CPT-2′-*O*-Me-cAMP is a specific activator of Epac [22] that potentiated the first peak of *I_SC_* to 140.3±7.2% (+8-CPT-2′-*O*-Me-cAMP: 24.54±1.26 µA/cm^2^, *p<0.05*, *n* = 4–6), but the second peak was not significantly potentiated. Sp-6-Phe-cAMP is a potent PKA activator. However, it does not activate Epac and thus can serve as an Epac negative control [23], [24]. Sp-6-Phe-cAMP potentiated the first peak of *I_SC_* but not the second peak. The first peak of *I_SC_* was increased to 128.1±6.6% of the control response (+Sp-6-Phe-cAMP: 22.42±1.15 µA/cm^2^, *p<0.05*, *n* = 4–6).

To test the inhibitory effect of different CysLT_1_ antagonists, montelukast, pranlukast, or zafirlukast (1 µM) were added together with various potentiators (1 µM) 10 min prior to the addition of UDP (100 µM). As summarized in Fig. 5F, the UDP-evoked *I_SC_* (first peak) in the presence of cAMP elevating agents (forskolin and 8-Br-cAMP), Epac (8-CPT-2′-*O*-Me-cAMP) or PKA (Sp-6-Phe-cAMP) activator was suppressed by different CysLT_1_ antagonists. Pranlukast inhibited the effects of forskolin (43.7±6.0% of control), 8-Br-cAMP (42.1±6.1% of control), and 8-CPT-2′-*O*-Me-cAMP (33.6±9.6% of control). Zafirlukast inhibited the effect of 8-Br-cAMP (63.0±5.3% of control) and Sp-6-Phe-cAMP (70.3±4.2% of control). Montelukast did not possess any inhibitory effects. None of the CysLT_1_ antagonists inhibited the second peak of *I_SC_* (data not shown). Taken together, pranlukast may inhibit the UDP-induced first peak *I_SC_* mediated by Epac, while zafirlukast is likely to inhibit the effect mediated by PKA. Montelukast, however, does not exert similar inhibitory action.

### Pranlukast, but not montelukast or zafirlukast, inhibited the activation of Epac

**Figure 6:**
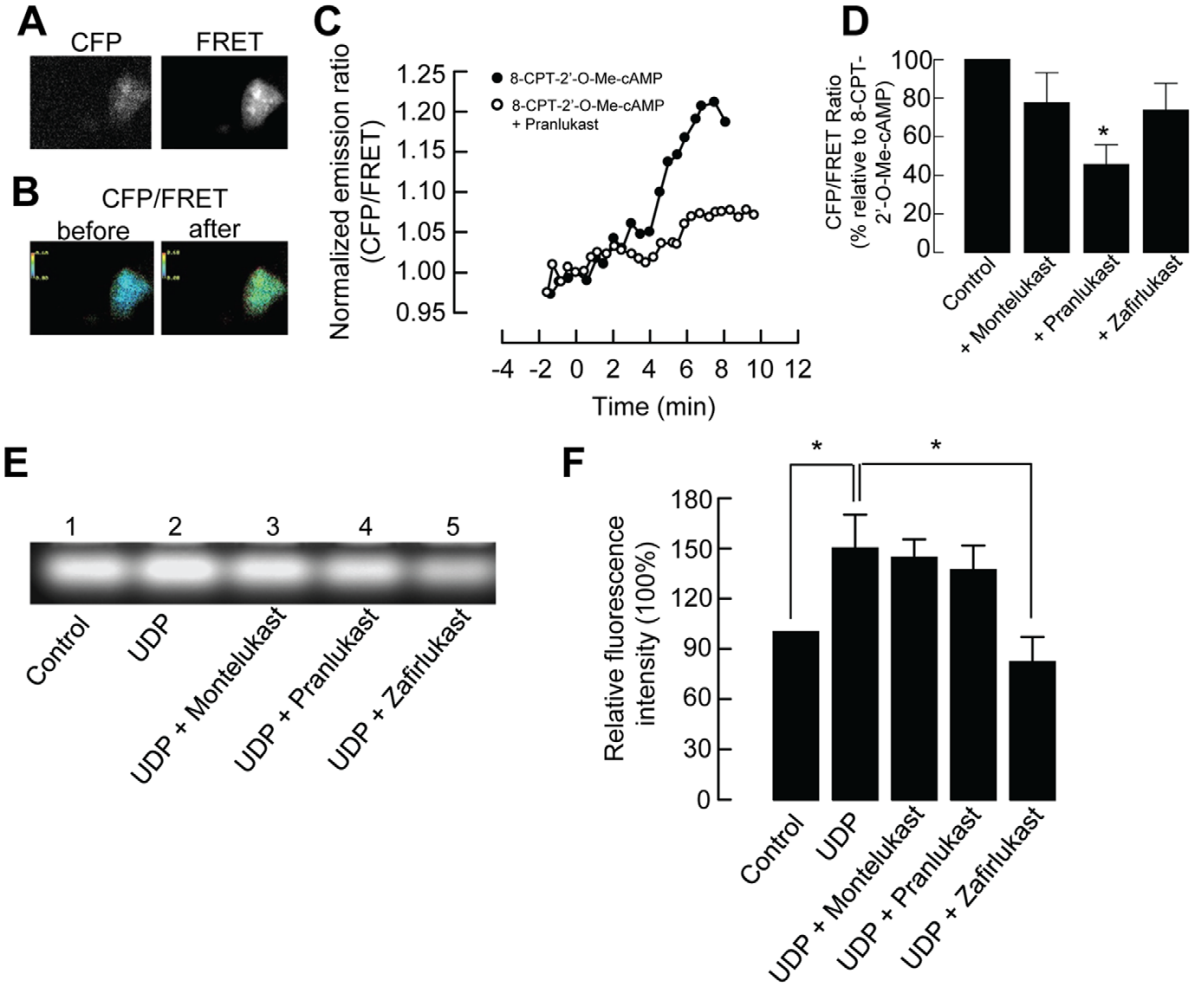
Effects of CysLT_1_ receptor antagonists on Epac activation and PKA activity. (A) The monochrome CFP and FRET images showing the cytosolic distribution of the fluorescent Epac probe in 16HBE14o- cells transfected with CFP-Epac-YFP. (B) Representative pseudocolor images of CFP/FRET emission ratios before and after the addition of 8-CPT-2′-*O*-Me-cAMP. (D) Real-time cAMP changes (normalized CFP/FRET emission ratio) recorded in cells stimulated with 50 µM 8-CPT-2′-*O*-Me-cAMP with or without 1 µM pranlukast shown in (B). The agents were added at time zero. (C) Summarized data showing the effect of CysLT_1_ receptor antagonists on the CFP/FRET emission ratio. Each column represents the mean ± S.E. (*n* = 8–10). (*, *p*<0.05, Student's *t*-test compared with control). (E) Confluent 16HBE14o- cells were treated with either vehicle alone (control), 100 µM UDP, or UDP with different CysLT_1_ receptor antagonists (1 µM) for 5 min. PKA activity was measured as a function of fluorescence intensity. (F) Summarized data showing the relative fluorescence level as compared with the control level. Each column represents the mean ± S.E. (*, *p*<0.05, *n* = 4, one-way ANOVA with Bonferroni post-hoc test).

To examine whether the inhibitory action of pranlukast occurs through Epac, we monitored Epac1 activation by using a cyan fluorescent protein (CFP)-Epac-yellow fluorescent protein (YFP) fusion construct, which has been used by others to detect real-time changes in [cAMP] via a fluorescence resonance energy transfer (FRET)-based approach [25]–[27]. Fig. 6A shows that 16HBE14o- cells were successfully transfected with the fusion construct of CYP-Epac-YFP, which is sensitive to the dynamic changes of [cAMP]. As expected, the Epac activator 8-CPT-2′-*O*-cAMP (50 µM) evoked about a 20% increase in the CFP/FRET emission ratio, which indicates a global increase in cAMP (Fig. 6B and C). Pranlukast could inhibit the activation of Epac mediated by 8-CPT-2′-*O*-cAMP. Fig. 6D summarizes the inhibitory effects of montelukast, pranlukast, and zafirlukast. In the presence of pranlukast (1 µM), the increase in the CFP/FRET emission ratio induced by 8-CPT-2′-*O*-cAMP was reduced to 45.5% of the control (*p*<0.05, *n* = 8). Montelukast (1 µM) and zafirlukast (1 µM) exhibited no inhibitory effects (*p*>0.05, *n* = 8–10). In the control experiments, addition of montelukast, pranlukast or zafirlukast (1 µM) did not induce any discernible increase in FRET ratio (*n* = 3).

### Zafirlukast, but not montelukast or pranlukast, inhibited UDP-activated PKA activity

The cAMP-dependent pathway underlying the inhibitory effects of montelukast, pranlukast, and zafirlukast on UDP was also studied by a PKA activity assay. PKA activity in 16HBE14o- cells was measured by a PKA assay that measures the phosphorylation of Kemptide, a synthetic substrate specific for PKA. The PKA activity was measured as a function of fluorescence intensity. As shown in Fig. 6E, phosphorylated peptides migrated towards the positively charged anode. The application of 100 µM UDP for 5 min significantly increased PKA activity by 150.0±20.2% when compared with that in untreated cells. The UDP-induced PKA activity was suppressed after pretreatment with zafirlukast (1 µM) to 82.0±15.1% (*p<0.05*, *n* = 4), whereas montelukast (1 µM) and pranlukast (1 µM) did not significantly inhibit UDP-induced PKA activity. Summarized data are shown in Fig. 6F. Addition of montelukast, pranlukast, zafirlukast, LTC_4_ or LTD_4_ (1 µM) did not significantly affect the PKA activity of the 16HBE14o- cells when compared with untreated control (one-way ANOVA, *n* = 3, *p*>0.05).

### Expression of Epac in 16HBE14o- cells

**Figure 7:**
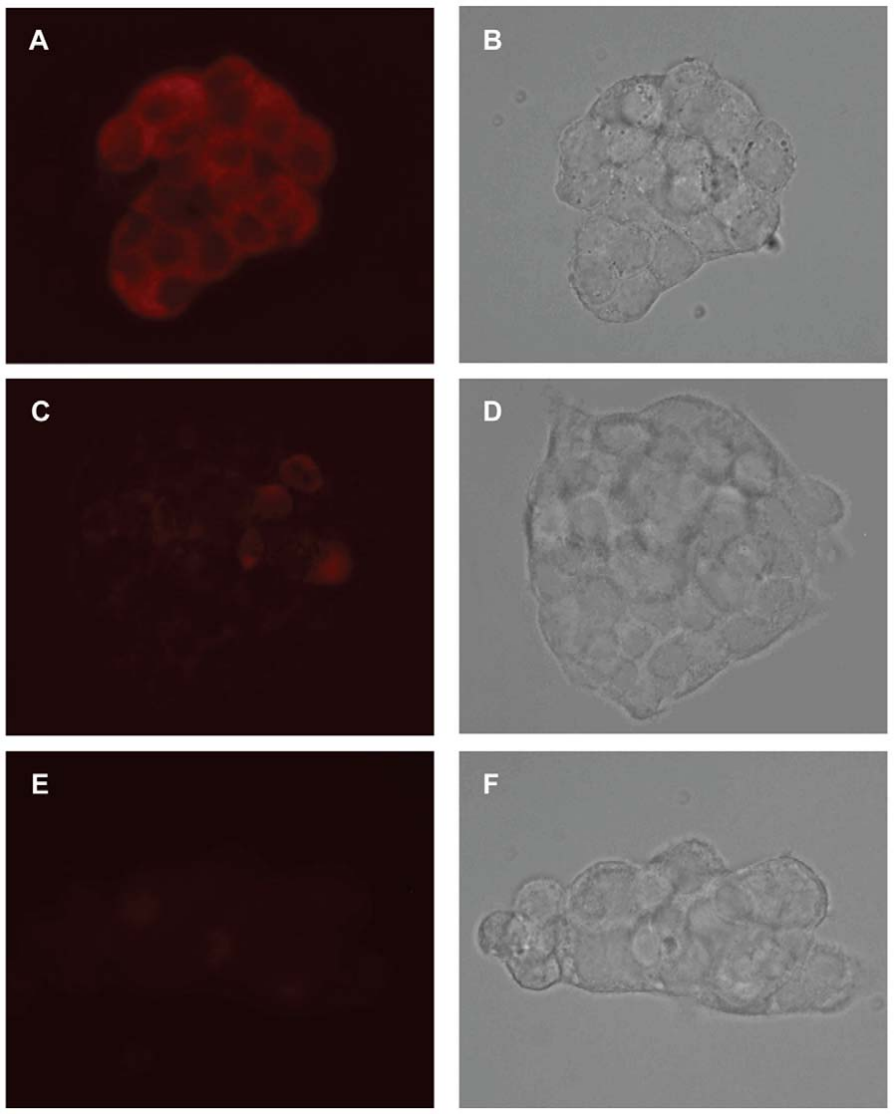
Immunolocalization of Epac 1 and Epac 2 in 16HBE14o- cells. With the use of specific antibodies, immunofluorescence staining of Epac 1 (A) and Epac 2 (C) were localized to the cytoplasm of 16HBE14o- cells, as indicated by the red colour. Under the conditions specified in the Methods section, the signal intensity of Epac 1 was stronger than that of Epac 2, which may suggest a higher and more uniform expression of Epac 1 in these cells. In the negative control (E), omission of the primary antibodies resulted in a complete loss of the immunofluorescence. The corresponding images taken in brightfield were shown in B, D and F.

As our data suggest pranlukast inhibited the activation of Epac, we further studied the distribution of endogenous Epac 1 and Epac 2 in 16HBE14o- cells by immunostaining (Fig. 7). Epac 1 staining (red) of 16HBE14o- cells was mostly localized to the cytoplasm of 16HBE14o- cells. The signal intensity of Epac 1 was stronger than that of Epac 2, which may suggest a higher and more uniform expression of Epac 1 in these cells. In the control experiment, omission of the primary antibodies resulted in a complete loss of the immunofluorescence.

## Discussion

### Crosstalk between CysLT_1_ receptor and P2Y_6_ receptors

In the airway, Cl^−^ secretion and Na^+^ reabsorption can be modulated by the activation of multiple P2Y receptors. The coordinated regulation of Cl^−^ secretion and Na^+^ reabsorption is necessary to maintain the thickness and composition of airway surface liquid, which in turn affects airway mucus clearance [28]. We have demonstrated previously that 16HBE14o- cells express multiple P2Y receptors, including P2Y_1_, P2Y_2_, P2Y_4_, and P2Y_6_. When activated by UDP, P2Y_6_ receptors are coupled to Ca^2+^- and cAMP-dependent pathways, thereby leading to transepithelial Cl^−^ secretion [11]. Recently, it was discovered that crosstalk exists between P2Y_6_ and CysLT_1_ receptors [6], [29]. CysLT_1_ is the receptor of CysLTs, which are key mediators of airway inflammatory disorders. Both P2Y and CysLT_1_ receptors belong to the superfamily of G protein-coupled receptors (GPCRs), and previous studies have suggested functional and structural similarities between these two receptors [30]. CysLT_1_ receptor antagonists inhibit the effects of nucleotides acting at P2Y receptors in monocyte-macrophage-like cell lines [10]. CysLT_1_ receptor antagonists inhibit UDP- but not carbachol-induced [Ca^2+^]_i_ increases, suggesting that the antagonists specifically interact with the P2Y receptor signaling pathway. However, the detailed molecular mechanism underlying the inhibition remains unresolved. More importantly, whether similar inhibitory effects could be observed in human airway epithelia remains unknown. In the present study, the effects of three specific CysLT_1_ antagonists on P2Y_6_ receptor-mediated signaling pathways and ion transport in human bronchial epithelial 16HBE14o- cells were examined.

In this study, the protein expression of P2Y_6_ and CysLT_1_ receptors in 16HBE14o- cells was demonstrated by western blotting. Activation of apical P2Y_6_ receptors by UDP elicited a concentration-dependent increase in both *I_SC_* and [Ca^2+^]_i_, confirming their role in stimulating transepithelial Cl^−^ secretion in human airway epithelia [11], [18], [31]. However, stimulation of the CysLT_1_ receptors with LTC_4_ or LTD_4_ failed to elicit any receptor-mediated [Ca^2+^]_i_ or *I_SC_* increases. The CysLT_1_ receptor is primarily coupled to G_q/11_ protein, which leads to activation of the PLC/IP_3_ pathway with intracellular Ca^2+^ mobilization in different cell types [30]. In cells that express both types of receptors, the addition of LTD_4_ and UDP caused a robust increase in [Ca^2+^]_i_ [7], and LTC_4_ could cross-desensitize UDP receptors [6]. In addition, 16HBE14o- cells possess an active biosynthetic pathway for the production of leukotrienes, such as LTC_4_ [32]. Profita et al. have shown that 16HBE14o- cells expressed functional CysLT_1_ receptors that coupled to the STAT-1 signaling pathway. The authors, however, had to enhance the expression of CysLT_1_ receptor by transfecting the cells with a construct encoding the receptor to circumvent the low and variable receptor expression in the cells [33]. Therefore, the fact that no CysLT_1_ receptor-mediated [Ca^2+^]_i_ response was observed in this study may be related to the low-level expression of the receptor, or the receptor may be coupled less efficiently to G_q/11_ protein. Moreover, the CysLT_1_ receptor agonists (LTC_4_ and LTD_4_) and antagonists (montelukast, pranlukast and zafirlukast) at 1 µM had no effect on either FRET ratio or PKA activity. These data further suggest that 16HBE14o- cells do not possess functional CysLT_1_ receptors. However, we could not exclude the possibility that the CysLT_1_ receptor may still couple to other cellular signaling pathways without interfering with the P2Y_6_ receptor -mediated Ca^2+^ and *I_SC_* responses.

### CysLT_1_ antagonists inhibited UDP-induced *I_SC_*

To examine whether the CysLT_1_ antagonists would have any effect on P2Y_6_ receptor-mediated ion transport processes, the effects of three commonly used CysLT_1_ antagonists, montelukast, pranlukast, and zafirlukast, on UDP-induced *I_SC_* were examined. They are potent and selective CysLT_1_ receptor antagonists marketed for the treatment of asthma [34] and are sold under the trade names of Singulair, Ultair, and Accolate, respectively [35]. The cells were pretreated with CysLT_1_ antagonists 10 min before addition of UDP. In general, all three CysLT_1_ antagonists inhibited the first peak of UDP-induced *I_SC_*, with montelukast inhibiting the peak at a lower concentration than that of pranlukast and zafirlukast. However, the second *I_SC_* peak was not influenced by these antagonists. Therefore, all three CysLT_1_ receptor antagonists, when applied to the apical but not basolateral side of the epithelia, are capable of inhibiting the transient but not the sustained component of the *I_SC_*, resulting in the inhibition of Cl^−^ secretion across the airway epithelia.

### Montelukast inhibited UDP-induced [Ca^2+^]_i_

Recently, we have demonstrated that P2Y_6_ receptors couple to both cAMP- and Ca^2+^-dependent pathways in 16HBE14o- cells [11]. For the Ca^2+^-dependent pathway, upon the coupling of UDP to P2Y_6_ receptors, there is an elevation of [Ca^2+^]_i_, which is caused by the activation of PLC and thus an increased production of IP_3_. In our experiments, upon the addition of UDP, there was a concentration-dependent increase in [Ca^2+^]_i_. Unlike the biphasic *I_SC_* response, the UDP-induced [Ca^2+^]_i_ response is monophasic. For the [Ca^2+^]_i_ measurements, the cells were stimulated with UDP in a Ca^2+^-free solution to remove the complication and involvement of Ca^2+^ influx. The results demonstrated that only montelukast inhibited the UDP-evoked [Ca^2+^]_i_ increase, indicating that montelukast interferes with UDP-coupled calcium mobilization. Our data are partly consistent with those of a previous study showing similar inhibition by montelukast in a human monocyte-like cell line [10]. However, in our study, pranlukast did not cause any concentration-dependent inhibition of [Ca^2+^]_i_ change. Therefore, the inhibitory effect of CysLT_1_ antagonists on P2Y_6_-coupled calcium signaling pathway may be cell type-specific. Additionally, the inhibitory effect appears to be specific to the P2Y_6_ receptor, as montelukast failed to inhibit the P2Y_2_/P2Y_4_ receptor-mediated increases in [Ca^2+^]_i_ and *I_SC_*. The cellular mechanism underlying the effect of montelukast was further investigated in 16HBE14o- cells treated acutely with pharmacological agents inhibiting the P2Y_6_-PLC-IP_3_-Ca^2+^-signaling cascade at different levels. For the first series of experiments, the most downstream target, IP_3_ receptors, was inhibited by XeC, a novel blocker of the IP_3_ receptor [17]. In the presence of XeC, the UDP-induced [Ca^2+^]_i_ increase was inhibited. However, coincubation of a low concentration of XeC and montelukast did not cause an additional inhibition of the UDP-induced [Ca^2+^]_i_ increase when compared with the inhibition caused by montelukast alone. The data suggest that montelukast may interfere with the pathway at a level upstream of the IP_3_-induced Ca^2+^ release. U73122, a specific inhibitor of PLC [16], also reduced the UDP-induced [Ca^2+^]_i_ increase. Similarly, coincubation of a low concentration of U73122 and montelukast failed to induce additional inhibition of the UDP-induced [Ca^2+^]_i_ increase when compared with the inhibition caused by montelukast alone. The data rule out the possible interference of montelukast on PLC activity. Moreover, our data showed that only montelukast applied at the apical but not basolateral compartment could inhibit the [Ca^2+^]_i_ increase induced by UDP. Therefore, montelukast is likely to interfere with the pathway at a level upstream of PLC activity and the production of IP_3_, probably at the receptor level. It has been proposed that the functional inhibition of nucleotide-elicited effects by leukotriene antagonists was mediated through direct allosteric interaction with the P2Y receptor [10], which is phylogentically related to CysLT receptors [36]. However, the detailed molecular interaction remains unknown and requires further investigation. Another possibility is that the CysLT_1_ antagonists exerted their inhibitory effects via an as yet unidentified signaling mechanism common to P2Y_6_ receptors, but not characteristic of other P2Y receptor subtypes.

### Pranlukast and Zafirlukast inhibited UDP-induced *I_SC_* via different mechanisms

Airway epithelial Cl^−^ secretion is stimulated by two important cytosolic second messengers, [Ca^2+^]_i_ and cAMP, and the latter could also potentiate Ca^2+^-mediated Cl^−^ secretion in intestinal T84 cells [37]. PKA and Epac are two downstream targets of cAMP that transduce diverse cellular actions [12], [38]. In intestinal epithelial cells, cAMP/PKA may potentiate Cl^−^ secretion by phosphorylating apical cystic fibrosis transmembrane conductance regulator (CFTR) Cl^−^ channels. Other potential targets include basolaterally located Na^+^/2Cl^−^/K^+^ cotransporters or cAMP-dependent K^+^ channels [39]. The effect of Epac on epithelial Cl^−^ secretion or ion channel activity is less well understood. A recent study suggested that Epac could regulate intestinal Cl^−^ secretion via a PKA-independent mechanism by stimulating the PLC/IP_3_-mediated calcium signaling pathway in intestinal epithelial T84 cells [21]. However, we were not able to detect any increase in [Ca^2+^]_i_ induced by Epac activators (Ko et al., unpublished data). The addition of forskolin also could not stimulate any increase in [Ca^2+^]_i_ in 16HBE14o- cells [11]. In this study, we investigated whether the three CysLT_1_ receptor antagonists interfered with the potentiating effects of cAMP and Epac on UDP-mediated Cl^−^ secretion. Cells were treated with drugs activating the adenylate cyclase-cAMP-PKA/Epac signaling cascade at different levels. Firstly, two general cAMP level elevators, forskolin and 8-Br-cAMP, were used. Forskolin is an adenylyl cyclase activator, while 8-Br-cAMP is a cAMP analog; therefore, both could increase the intracellular level of cAMP. The first and second peaks of the *I_SC_* response were potentiated by forskolin and 8-Br-cAMP. Our data demonstrated that pranlukast and zafirlukast inhibited the first peak of the UDP-induced *I_SC_* potentiated by forskolin or 8-Br-cAMP. However, montelukast did not inhibit the first peak of potentiated *I_SC_*. On the other hand, the second peak of the potentiated *I_SC_* was not inhibited any of the three CysLT_1_ antagonists. The data demonstrated that the the UDP-evoked Cl^−^ secretion potentiated by the presence of various cAMP elevating agents was inhibited by pranlukast and zafirlukast but not montelukast.

As mentioned above, cAMP has two downstream targets, PKA and Epac, which cannot be distinguished by general cAMP level elevators. Thus, 8-CPT-2′-*O*-Me-cAMP, a specific activator of Epac [22], was used to investigate the effect of CysLT_1_ antagonists on Epac-potentiated *I_SC_* induced by UDP. 8-CPT-2′-*O*-Me-cAMP potentiated the first peak but not the second peak of the UDP-induced *I_SC_*. The 8-CPT-2′-*O*-Me-cAMP-potentiated first peak of UDP-induced *I_SC_* was inhibited only by pranlukast. It appears that pranlukast could reduce the *I_SC_* response potentiated through the action of Epac. There are two possibilities for the inhibitory mechanism. Pranlukast may inhibit the UDP-induced *I_SC_* through inhibition of cAMP production or Epac activation. However, it is unlikely that pranlukast inhibits the production of cAMP because pranlukast also inhibited the UDP-induced *I_SC_* potentiated by the general cAMP analog 8-Br-cAMP. To confirm that pranlukast reduces the *I_SC_* response by acting through Epac, FRET was employed. In the study, we used a FRET-based cAMP indicator, CFP-Epac-YFP. 8-CPT-2′-*O*-Me-cAMP activated Epac, which caused a significant increase in the FRET emission ratio. In the presence of pranlukast, the increase in the FRET emission ratio elicited by 8-CPT-2′-*O*-Me-cAMP was reduced, indicating that pranlukast inhibited the activation of Epac. These data further confirms that pranlukast reduces UDP-evoked responses by acting through the action of Epac, which is expressed in 16HBE14o- cells as shown by immunocytochemistry. The expression of Epac 1 but not Epac 2 in 16HBE14o- cells is consistent with the observation that Epac 1 mRNA is expressed ubiquitously, whereas Epac 2 mRNA is predominately expressed in the brain and endocrine tissues [40].

On the other hand, Sp-6-Phe-cAMP served as an Epac-negative control because it activates PKA but not Epac [23], [24]. Sp-6-Phe-cAMP also potentiated the first peak but not the second peak of UDP-induced *I_SC_*. The UDP-induced *I_SC_* first peak potentiated by Sp-6-cAMP was inhibited by zafirlukast, but not by montelukast or pranlukast. The cAMP-dependent PKA pathway underlying the effects of montelukast, pranlukast, and zafirlukast on UDP was further studied by a PKA activity assay. The data showed that UDP-induced PKA activity was suppressed only in the presence of zafirlukast, confirming that zafirlukast could reduce the *I_SC_* response acting through the action of PKA. Taken together, these findings imply that montelukast, pranlukast, and zafirlukast differentially inhibited the second messenger-mediated changes in *I_SC_* induced by the apical application of UDP. In particular, pranlukast could inhibit the cAMP-dependent pathway potentiated through the action of Epac, while zafirlukast may inhibit the same pathway potentiated through PKA. Montelukast, however, does not interfere with the cAMP-dependent pathway. However, we could not exclude the possibility that the CysLT_1_ antagonists may have direct effects on apical Cl^−^ channels, which await further investigation.

### P2Y_6_ receptors and inflammatory response

Aside from regulation of ion transport in various epithelia, P2Y receptors, including P2Y_6_, have been implicated as important players in the initiation, amplification, and spread of acute inflammation as well as the downregulation of chronic inflammation [41], [42]. Recently, it was shown for the first time that P2Y_6_ receptors modulate the release of the chemokines CCL20 and IL-8 from human nasal epithelial cells and thereby could alter immune cell recruitment in airways [43]. The specific agonist for P2Y_6_ receptors, UDP, stimulates the production of proinflammatory cytokines in retinal pigment epithelial cells [44] and monocytic cells [45]. In an ulcerative colitis mouse model, both P2Y_2_ and P2Y_6_ receptor mRNA and protein expression was upregulated in the inflammatory response of intestinal epithelial cells. Stimulation of the intestinal epithelial cells with the P2Y_6_ receptor agonist UDP resulted in an increased expression and release of IL-8 [46]. Montelukast, pranlukast, and zafirlukast are now widely used clinically for the treatment of asthma and allergic rhinitis. Our data suggest that they may act through a mechanism of action that is independent of CysLT_1_ receptor antagonism. Therefore, it is possible that part of the antiinflammatory effect of CysLT_1_ receptor antagonists in asthma is mediated through the P2Y_6_-mediated signaling pathway, hence ameliorating the proinflammatory responses. In airway epithelia, it is important to maintain the volume or height of the liquid (air surface liquid, ASL) that lines the surface of airway epithelia. Enhancing fluid and electrolyte transport may improve airway surface hydration and improve mucus clearance, which is hypersecreted in various respiratory diseases, such as asthma. Stimulation of various P2Y receptors, including P2Y_6_ receptors, will increase Cl^−^ secretion into the lumen of the airway, which subsequently drives water secretion due to the high water permeability of the airway epithelia.Therefore, the inhibition of P2Y_6_ receptor-mediated Cl^−^ secretion may have the opposite effect by reducing the hydration of the airway surface, which therefore compromises the innate immunity of the airway epithelia.

In summary, our data demonstrate that the three specific CysLT_1_ receptor antagonists have different pharmacological actions against the P2Y_6_ receptor-mediated signaling pathway in 16HBE14o- cells. Although all antagonists inhibited the UDP-evoked *I_SC_* increase, only montelukast inhibited Ca^2+^ signaling, while pranlukast and zafirlukast inhibited the potentiation effect of cAMP signaling mediated by Epac and PKA, respectively. The data showed that these three specific CysLT_1_ receptor antagonists are not as highly specific as previously thought, and this may provide implications for the clinical use of these compounds in asthma or other inflammatory conditions. Therefore, further studies should be undertaken to evaluate the efficiency and underlying cellular mechanisms of the therapeutic rationale. With respect to this study, the detailed mechanisms of CysLT_1_ antagonists on inflammation-related P2Y receptors should be further investigated to uncover the crossregulatory mechanisms between CysLT_1_ and P2Y receptors, and more importantly, the impact on airway epithelial transport.

## Materials and Methods

### Cell Culture

All experiments were performed using the immortalized cell line 16HBE14o-, which was derived from bronchial surface epithelial cells [47]. Standard culture techniques were used as described previously [11], [48]. In brief, cells were maintained in Minimum Essential Medium with Earle's salt supplemented with 10% (v/v) fetal bovine serum, 1% (v/v) l-glutamine, 100 IU/ml penicillin, and 100 µg/ml streptomycin. Cells were cultured on plastic flasks coated with fibronectin and collagen (BD Biosciences, San Jose, CA) and were incubated in humidified 95% air-5% CO_2_ at 37°C.

### Western Blot

Cells grown in culture flasks were lysed in radioimmunoprecipitation assay buffer [1% NP-40, 0.1% sodium dodecyl sulfate, 0.5% deoxycholic acid, 50 mM Tris-HCl (pH 7.4), 150 mM NaCl] supplemented with protease inhibitors [5 µg/ml aprotinin, 1 µg/ml leupeptin, 1 mM phenylmethylsulfonyl fluoride, 1 mM sodium fluoride, 1 mM sodium orthovanadate, 2 mM β-glycerolphosphate, 1 mM ethylene glycol tetraacetic acid, 1 mM ethylenediaminetetraacetic acid]. The cell lysate was collected, and the supernatant was harvested after centrifugation at 20,000×*g* for 20 min at 4°C. The total protein content in each sample was determined using the Bradford assay (Bio-Rad, Hercules, CA). Forty micrograms of protein were used for western blotting and separated by 10% sodium dodecyl sulfate polyacrylamide gel electrophoresis. Separated proteins were electroblotted to a polyvinylidene fluoride membrane (Immobilon-P, Millipore Corporation, Billerica, MA) by using a wet transfer system (Bio-Rad). Membranes were blocked for 1 h at room temperature by using 1% bovine serum albumin in phosphate buffered saline containing 0.05% Tween 20 and incubated overnight at 4°C with specific CysLT_1_ receptor polyclonal antibody (1∶300, rabbit polyclonal anti-CysLT_1_, no. 120500, Cayman Chemical, Ann Arbor, MI) or P2Y_6_ receptor antibody (1∶200, no. APR-011, rabbit polyclonal anti-P2Y_6_, Alomone, Jerusalem, Israel). The positions of positive bands were detected by incubation with horseradish peroxidase-conjugated anti-rabbit immunoglobulin G (Dako, Glostrup, Denmark) and visualized using an enhanced chemiluminescence detection system (Amersham Biosciences, Piscataway, NJ). Their apparent molecular masses were calculated based on prestained sodium dodecyl sulfate polyacrylamide gel electrophoresis low-range protein standards (Bio-Rad).

### Immunocytochemistry

16HBE14o- cells cultured on 12-mm glass coverslips were rinsed twice with phosphate-buffered saline (PBS) and then fixed in a 4% (w/v) paraformaldehyde solution for 30 min at 4°C. Cells were permabilized with 0.5% Triton X-100 in PBS for 15 min and blocked with 10% fetal calf serum (FCS) in PBS for 30 min at room temperature. Cells were then incubated overnight at 4°C with Epac 1 or Epac 2 rabbit polyclonal antibodies (Abcam: ab21236 or ab21238) at a dilution of 1∶200 in the blocking solution (10% FCS in PBS). At the end of primary antibody incubation, cells were washed three times in PBS and then incubated with goat anti-rabbit IgG (H+L) coupled to Alexa Fluor® 568 (Invitrogen: A11011) at 1∶400 dilution in PBS for 15 min. After rinsing off the excess fluorescent dye-labeled secondary antibody, the coverslip was mounted on a microscopic slide using Shandon Immu-Mount™ (Thermo Scientific) and visualized with a Zeiss Axioskop 2 plus microscope under fluorescent microscopy and brightfield. Control staining was performed following the above procedure and differed only in the omission of the primary antibodies.

### Measurement of short-circuit current (*I_SC_*)

Confluent 16HBE14o- cells were used to measure *I_SC_* as described previously [37]. The monolayers were cultured on Transwell-COL membranes (Costar, Cambridge, MA) with a 0.4-µm pore diameter (culture area 0.2 cm^2^), mounted in an Ussing chamber, and bathed in normal Krebs-Henseleit (K-H) solution. To generate a favorable gradient for Cl^−^ exit, a basolateral-to-apical Cl^−^ gradient was applied across the monolayers by changing the apical K-H solution to one with a reduced Cl^−^ concentration [47]. The potential difference was clamped to 0 mV, and *I_SC_* was simultaneously measured using a voltage-clamp amplifier (VCC MC6; Physiologic Instruments, San Diego, CA). A transepithelial potential difference of 1 mVs was applied periodically, and the resultant change in the current was used to calculate the transepithelial resistance using Ohm's law. Cells reached confluence after 10 days with a resistance greater than 150 Ω·cm^2^.

### Measurement of PKA activity

Confluent 16HBE14o- cells grown on 6-well plates were incubated with vehicle alone or UDP in the presence or absence of different CysLT_1_ receptor antagonists for 5 min. The PKA activity was assayed using the PepTag® non-radioactive cAMP-dependent protein kinase assay system (Promega, Madison, WI) as described previously [37]. The phosphorylated and nonphosphorylated samples were separated on a 0.8% agarose gel at 100 V for 15 min. The gel was photographed, and the fluorescence intensity was quantified by the FluorChem™ 8000 imaging system.

### Measurement of intracellular calcium concentration ([Ca^2+^]_i_)

Calcium signals in cells grown on glass coverslips were measured as previously described [11], [49]. Briefly, cells were loaded with Fura-2 by incubation (45 min, 37°C) in K-H solution containing 3 µM Fura-2-AM and 1.6 µM pluronic F127. The Fura-2 loaded cells were washed with K-H solution, and the entire coverslip was then transferred to a closed perfusion chamber mounted on an inverted microscope (Olympus IX70, Center Valley, PA) equipped with a ×20 water immersion objective (numerical aperture 0.6). The excitation light source was provided by a multi-wavelength illuminator (Polychrome IV, TILL Photonics, GmBH, Hilden, Germany) that enables rapid interchange between two excitation wavelengths (340 and 380 nm). The emitted fluorescence was passed through a 400-nm dichroic mirror, filtered at 510 nm, and then collected using a digital cooled CCD camera (Quantix, Photometrics, Tucson, AZ). Images were digitized and analyzed using MetaFluor Imaging Software (v.7.5, Molecular Devices, Downingtown, PA). Fura-2 ratios were used to represent changes in [Ca^2+^]_i_.

### Real-time monitoring of cAMP by FRET

Real-time cAMP changes in living cells were monitored using CFP-Epac-YPF, an Epac-based polypeptide FRET reporter [27]. FRET imaging experiments were performed using the MetaFluor Imaging System (with FRET module) similar to that described above. 16HBE14o- cells were transfected with the Epac-based cAMP sensor. Cells on glass coverslips were placed on the inverted microscope and excited at 436 nm. CFP and YFP images were simultaneously recorded by the imaging setup equipped with the Photometrics DV^2^ emission splitting system (Photometrics, Tucson, AZ) including two emission filters (470/30 nm for CFP and 535/30 m for FRET). Acquired fluorescence images were background subtracted, and real-time cAMP changes were represented by normalized CFP/FRET emission ratio similar to that described by Li et al. [50].

### Materials and solutions

The bicarbonate-buffered K-H solution contained the following components (in mM): NaCl, 117; NaHCO_3_, 25; KCl, 4.7; MgSO_4_, 1.2; KH_2_PO_4_, 1.2; CaCl_2_, 2.5; and d-glucose, 11; its pH was 7.4 when bubbled with 5% CO_2_/95% O_2_. The nominally Ca^2+^-free solution was prepared by omitting Ca^2+^ from the above solution. The low Cl^−^ solution (10 mM) was prepared by isosmotically replacing NaCl, KCl, CaCl_2_, and MgCl_2_ with Na-gluconate, K-gluconate, Ca-gluconate, and Mg-sulfate, respectively. The membrane-permeant acetoxymethylester (AM) forms of Fura-2 and pluronic F127 were obtained from Molecular Probes (Eugene, OR). UDP, forskolin, and 8-Br-cAMP were obtained from Sigma-Aldrich (St. Louis, MO, USA). Before the use of UDP, this nucleotide (10 mM) was incubated (1 h at 37°C) in 4-(2-hydroxyethyl)-1-piperazineethanesulfonic acid-buffered saline containing hexokinase (10 IU/ml, Boehringer, Mannheim, Germany) and 22 mM d-glucose to remove contaminating nucleotide triphosphates. The resulting solution was then aliquoted and stored at −20°C. Montelukast, pranlukast, and zafirlukast were purchased from Cayman Chemical. 8-CPT-2′-*O*-Me-cAMP and Sp-6-Phe-cAMP were obtained from BIOLOG (Bremen, Germany). All other general laboratory reagents were obtained from Sigma-Aldrich, and all cell culture reagents were obtained from Invitrogen (Carlsbad, CA, USA).

### Data analysis

Pooled data are presented as means ± standard errors (S.E.), and values of *n* refer to the number of experiments in each group. Experimentally induced changes (Δ) in the Fura-2 fluorescence ratio and *I_SC_* were quantified by measuring each parameter at the peak of a response and subtracting the equivalent values measured immediately prior to stimulation. Statistical comparisons between original data before normalization were performed using one-way analysis of variance with Bonferroni post-hoc test, with a *p*<0.05 considered significant.
